# Diagnosis of cancer in the South and North of Nigeria: duration and causes of delay

**DOI:** 10.1186/s12913-025-12707-8

**Published:** 2025-05-21

**Authors:** Olufunke Fayehun, Patricia Apenteng, Usman Aliyu Umar, Kudus Oluwatoyin Adebayo, Eme Owoaje, Jo Sartori, Omolara Popoola, Ujunwa Nnabuife, Abiola Oladejo, Oladoyin Odubanjo, Omobolaji Ayandipo, Akin-Tunde Odukogbe, David Irabor, Julius Ijitola, Abubakar Bala Muhammad, Imani Haruna, Abdulrazak Ajiya, Abdul Rasheed Suleiman, Ibrahim Danladi Muhammad, Natalia Adamou, Nasir Garba Abdullahi, Saminu Muhammad, Isah Tijjani, Tijjani Nasiru Nagwamutse, Shehu Usman Abdullahi, Lawal Shittu, Khadija Abdullahi Ado, Ashiru Aliyu Umar, Asiya Sufyan Bello, Ibrahim Adamu Yakasai, Akinyinka Omigbodun, Richard Lilford

**Affiliations:** 1 University of Ibadan, Box 4078, University of Ibadan Post, Ibadan, 200001 Oyo Nigeria; 2 University of Birmingham, Edgbaston, B15 2TT UK; 3 Aminu Kano Teaching Hospital, PMB 3452, Zaria Road, Kano, Nigeria; 4 Bayero University Kano, PMB 3011, Kano, Nigeria; 5 University of The Witwatersrand, 1 Jan Smuts Avenue, Johannesburg, South Africa; 6 Nigerian Academy of Science, PMB 1004, University of Lagos Post Office, Akoka-Yaba, Lagos, Nigeria; 7 University College Hospital, Queen Elizabeth Road, Ibadan, Oyo State Nigeria; 8 Yusuf Maitama Sule University, Kofar Kansakali, Kano, 700282 Kano Nigeria; 9 Murtala Muhammed Specialist Hospital, Kufar Mata Rd, Kano City, 700114 Kano Nigeria; 10 Ministry of Health, 4 Post Office Road, Kano, Nigeria

**Keywords:** Cancer, Delay, Diagnosis, Health system

## Abstract

**Introduction:**

Nigeria has a growing cancer burden, with late presentation and delayed diagnosis contributing to poor outcomes. We explored the durations and causes of the delay in the diagnosis of four common and treatable cancer types (breast, colorectum, head and neck, and uterine cervix) in Nigeria.

**Methods:**

Retrospective study based on interviews with cancer patients following the Aarhus framework for designing and reporting such studies. The study focused on the first two of WHO’s three main designated stages of cancer diagnosis: duration from symptom to presentation and presentation to histological diagnosis. Our hospital-based study involved 264 patients recruited from tertiary care facilities in the Northwestern (Kano) and Southwestern (Ibadan) regions of Nigeria. We obtained quantitative data to measure the duration of delay by stage, while interview data were collected to explore the causes of delay. We analysed the data by computing the median duration for the two stages of delay, and framework analysis was used to identify themes on the causes of delay.

**Results:**

The median time to receive a cancer diagnosis after noticing the first symptoms was 12 months (interquartile range 5 to 27 months), with head and neck cancer patients reporting the most prolonged (15-month) delay. Patients waited a median of 3 months (interquartile range 12 months) before presenting their first cancer symptom to a healthcare professional. The median time for patients to receive a cancer diagnosis after the first presentation of symptoms to a formal healthcare professional was 5 months (interquartile range 12 months). There was wide variance for all time intervals. Patients reported visiting a median of 3 health facilities before diagnosis in a formal hospital setting. Qualitative findings identified two main reasons patients reported delays in cancer pathway to care: patient-related factors and health system issues.

**Conclusion:**

Long delays were observed, and more than half the delay followed presentation to the local health sector.

**Supplementary Information:**

The online version contains supplementary material available at 10.1186/s12913-025-12707-8.

## Background

Cancer is responsible for about one in six deaths worldwide [1]. Low and middle-income countries (LMICs) have a growing cancer burden, with significantly higher age-adjusted cancer mortality than high-income countries [2]. The majority of people with cancer are in late stage when they receive a diagnosis and treatment in Sub-Saharan Africa [3]– for example, a median of 75% for breast cancer, according to a systematic review in 2016 [4]. Delay in treatment is associated with poor prognosis [5, 6].

Nigeria is a LMIC in sub-Saharan Africa with a population of 224 million, larger than the population of the UK, France and Germany combined. A study of hospital case notes of urban dwellers identified from the Nigerian Cancer registry with breast, uterine cervix, colorectum or prostate cancer showed that between two-thirds and three-quarters of patients present late (stages 3 or 4), with no recent improvement [7]. Cancer incidence is rising in Nigeria as people adopt new lifestyles [7], and the overall cancer incidence is 113.6 per 100 000 persons per year whilst the overall cancer mortaliy is 74.5 per 100 000 persons per year [8]. Less than 10% of Nigerians are enrolled in the National Health Insurance Scheme which provides limited coverage of healthcare services [9–11]. Consequently, most Nigerian patients experience financial hardship due to high out-of-pocket expenditures related to cancer care [11–13].

In response to the growing cancer burden, Health boards in Nigeria have invested in facilities for curative treatment for common cancers [14, 15]. However, to realise the benefits of these investments, it will be necessary to reduce the delay between first symptoms and treatment. In this study, we explored the durations and causes of the delay to the point of histological diagnosis of cancer in health facilities in Nigeria. We focused on four cancer types: (1) breast, (2) colorectum, (3) head and neck, and (4) uterine cervix. We select these cancers because they are common, tend to present early with specific symptom groups and can be cured if treated early.

## Methods

### Framework

We conducted an observational study based on interviews with patients after they had received a diagnosis of one of the above four cancer types. The protocol for this study can be found at Protocol exchange [16]. We followed the Aarhus framework [17] for design and reporting studies on early cancer diagnosis. This statement seeks to systematise questionnaires to reconstruct a patient’s pathway from the first symptom (body change) to treatment. This pathway can be divided into stages and sub-stages. The WHO [18] designates three main stages: (1) first symptom to presentation to the formal health system (i.e. an allopathic health provider); (2) presentation to histological diagnosis, and (3) diagnosis to treatment. WHO guidelines [19] recommends that the interval from symptom onset to initiation of treatment should generally be less than 90 days to reduce delays in care and optimize treatment outcomes. We use the term ‘delay’ to indicate the time between one event and another (e.g. between first symptom and presentation to the formal health system. We could not measure the final WHO stage, diagnosis to treatment in this study (see below). We, therefore, measured the first two stages above and identified the total number of clinics each person visited on the pathway to diagnosis.

### Setting

Two geopolitical regions of Nigeria - the Northwest (Kano) and Southwest (Ibadan) were purposively selected to sample patients of considerable ethnic, religious, and cultural diversity. Kano is the second largest city in Nigeria, with over 4 million people, who are predominantly Muslims of Hausa ethnicity. Ibadan is the third largest city in Nigeria, with a population of 3.7 million practising Christianity and Islam.

### Recruitment strategies

Participants were recruited from the University College Hospital Ibadan, and Aminu Kano Teaching Hospital and Murtala Muhammad Specialist Hospital in Kano. These are the only hospitals providing specialist care in the study cities and, therefore, constitute the final common pathway for treatment (or the decision that treatment is not advisable) for all but a small proportion of patients who can afford and choose private care.

The ideal point to recruit patients would be at the ‘critical point’ where patients either receive first-line treatment or the decision that the cancer is too advanced to treat. Attempting to recruit patients after treatment would likely miss patients who did not remain under hospital care to receive treatment. Yet, these are the very people we need to identify to avoid skewing the sample towards those with earlier presentations. To overcome this problem, we considered conducting interviews in the patients’ homes, including all patients who had reached the critical point. However, we were advised by local clinicians that this would be seen as intrusive and culturally inappropriate, especially in Kano. We, therefore, recruited people at a point where the initial approach could be made in the outpatient department following histological diagnosis.

Eligible participants were identified by the patient’s clinician, who requested assent (see below). Assenting patients were introduced to the non-clinical researcher, who requested consent. The study selected patients who (1) had a first histologically confirmed diagnosis of cancer of the breast, colorectum, head and neck, or uterine cervix, (2) were aged ≥ 18 years, and (3) could provide informed consent. Participants were invited to bring any accompanying relative or friend to the interview.

### Ethics, consent, and permissions

Ethics approvals for this study were obtained from UI/UCH Health Research Ethics Committee (Ref: UI/EC/23/0282), Aminu Kano Teaching Hospital Health Research Ethics Committee (Ref: SHREC/2023/3965) and Kano State Ministry of Health Research Ethics Committee (Ref: AKTH/EC/3562). The researchers adhered to international standards for conducting health research [20] and ensured that the rights and well-being of participants were protected during the data collection. The study approach separated ‘assent’ from ‘consent’. Assent covers the process by which the researcher clinician, a study team member, asks the patient whether they would be prepared to meet the field researcher to discuss participation in an interview and describe their journey to a point where the diagnosis has been made. After explaining and discussing the study with the patient, the field researcher requested verbal and written consent from patients who agreed to be interviewed. Patients who lacked the capacity to give informed consent, who had recurrent cancer, or who were acutely ill were not recruited into the study.

### Data collection

Data were collected between July 1 and December 31, 2023, across the three tertiary hospitals. The study set out to interview 60 patients per cancer type per site, (240 per site). Our sample size calculations were based on the precision with which we can estimate the quantiles of the delay (median, 75 th, or 90 th percentiles) for any particular group. A sample of 60 individuals will allow us to estimate the median delay to a precision of approximately +/- 5 days (95% confidence interval), and the 75 th and 90 th percentiles to +/- and 12 days, respectively. The instrument we developed to collect the quantitative and qualitative data is enclosed in the Supplementary File (Patient interview guide).

The study collected quantitative information to measure the duration of delay by stage. If a participant could not immediately and confidently recall one of the above crucial dates, we used a ‘calendar methodology’ [21] to help recall and minimise telescoping (the tendency to perceive distant events as being more recent than they are and vice versa). We shared a calendar with the participants and asked them to provide important dates in their personal lives, such as a family member’s marriage. We then elicited a best estimate for each crucial event about these personally significant events. Qualitative data were collected to explore patients’ experiences at each step of the diagnostic pathway. All data collection was face-to-face and the sessions were audio-recorded with the permission of the participant.

### Data analysis

All interviews were audio recorded, anonymised, and transcribed verbatim. Interviews conducted in local languages (Yoruba or Hausa) were transcribed in the original language then back-translated to English by six culturally sensitive research assistants (2 in Ibadan and 4 in Kano). Each transcript was then read and checked by an academic member of the research team.

Quantitative data analysis included: (1) computing the median and ranges for the above two stages of delay, and (2) recording the number of clinic visits made on the pathway from presentation to diagnosis. The qualitative data was analysed using framework analysis [22]. Emerging themes were identified, and a thematic framework was developed to code responses. These themes were further classified into two of the three contributing factors from the Aarhus framework: Patient Factors and Healthcare provider and system factors. The qualitative information on reasons for delays in stages in the pathway was interpreted based on discussion and consensus among the study team.

### Patient and public involvement

Cancer patient advocates and healthcare professionals in the two study sites were first engaged in the research process during the planning stage. They participated in a series of stakeholder engagement meetings in Ibadan and Kano, where they identified important issues affecting the patients, forming the basis of this study’s objectives. In addition, they contributed their perspectives on the study design and methodologies, particularly in contextualizing the research instruments, which informed the training of research assistants and improved the conduct of the research.

## Results

### Characteristics of participants

A total of 264 participants were interviewed across the selected three tertiary hospitals in Kano and Ibadan. Table 1 presents the characteristics of participants. One hundred and thirty-five patients in Ibadan and one hundred and twenty-nine in Kano participated in the study. 60% (158/264) of participants were aged ≤ 50 years. 71% (188/264) were female; 99% (77/78) of participants with cancer of the breast were female, whilst 44% of participants with cancer of the colorectum and 29% of participants with head and neck were female. While most participants had a formal education (206 of 264), few reported working at the time of the interview (104 of 264). Just 10 of 264 participants had health insurance for cancer treatment.

**Table 1:** Characteristics of participants

Characteristics	*n*	%
**Location**		
Ibadan (Southern Nigeria	135	51.1
Kano (Northern Nigeria)	129	48.9
**Type of cancer**		
Breast	78	29.5
Cervix	70	26.5
Colorectum	48	18.2
Head and Neck	68	25.8
**Sex of participants**		
Female	188	71.2
Male	76	28.8
**Age group (years)**		
18–30	28	10.6
31–40	48	18.2
41–50	82	31.1
51–60	54	20.5
61–70	35	13.3
Above 70	17	6.4
**Highest Education**		
No Formal	55	20.8
Primary	51	19.3
Secondary	74	28.0
Tertiary	84	31.8
**Currently working**		
Yes	104	39.4
No	160	60.6
**Healthcare payment plan**		
Self	65	24.6
Family and friends	181	68.6
Health insurance	10	3.8
All of the above	1	0.4
Not sure	7	2.7

### Duration of delay at different stages

**Fig. 1:**
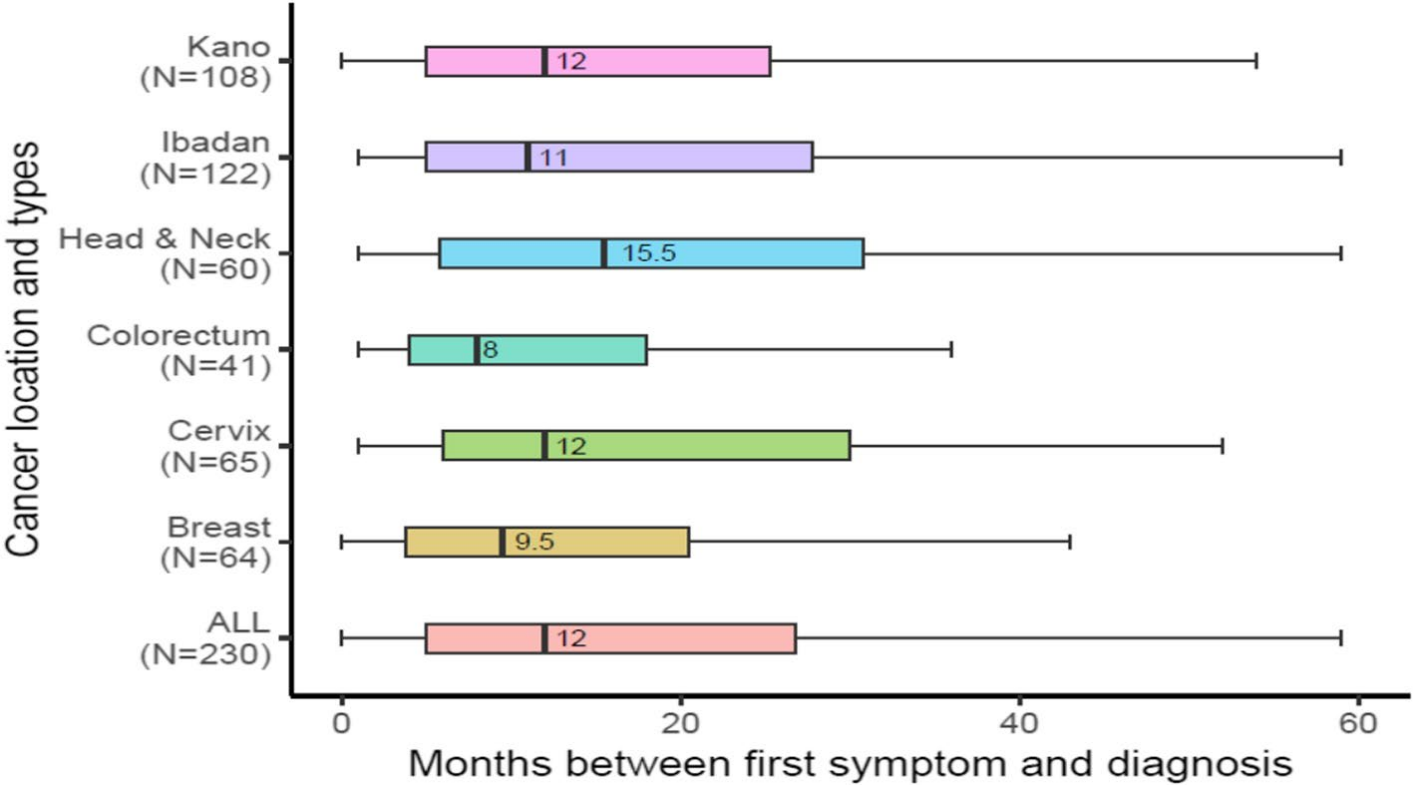
Duration of delay between first symptom and diagnosis, by site and by cancer type

The median total time to receive a cancer diagnosis after noticing the first symptoms was 12 months, with an interquartile range of 5 to 27 (Fig. 1). Patients with head and neck cancer experienced the most prolonged (15-month) delay (interquartile range 27 months). In contrast, colorectal cancer patients had the shortest duration, with a median of 8 months. The data were heavily skewed with patients waiting up to nearly 60 months.

**Fig. 2:**
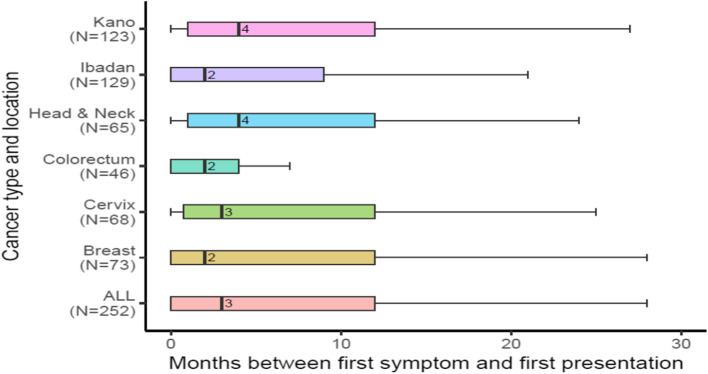
Duration of delay between first symptom and first presentation to a formal healthcare professional

Patients waited a median of three months before presenting their first cancer symptom to a healthcare professional (Fig. 2). This duration varied by study site and the type of cancer, with patients with cervix and head and neck cancer presenting later than those with breast and colorectum cancer.

**Fig. 3:**
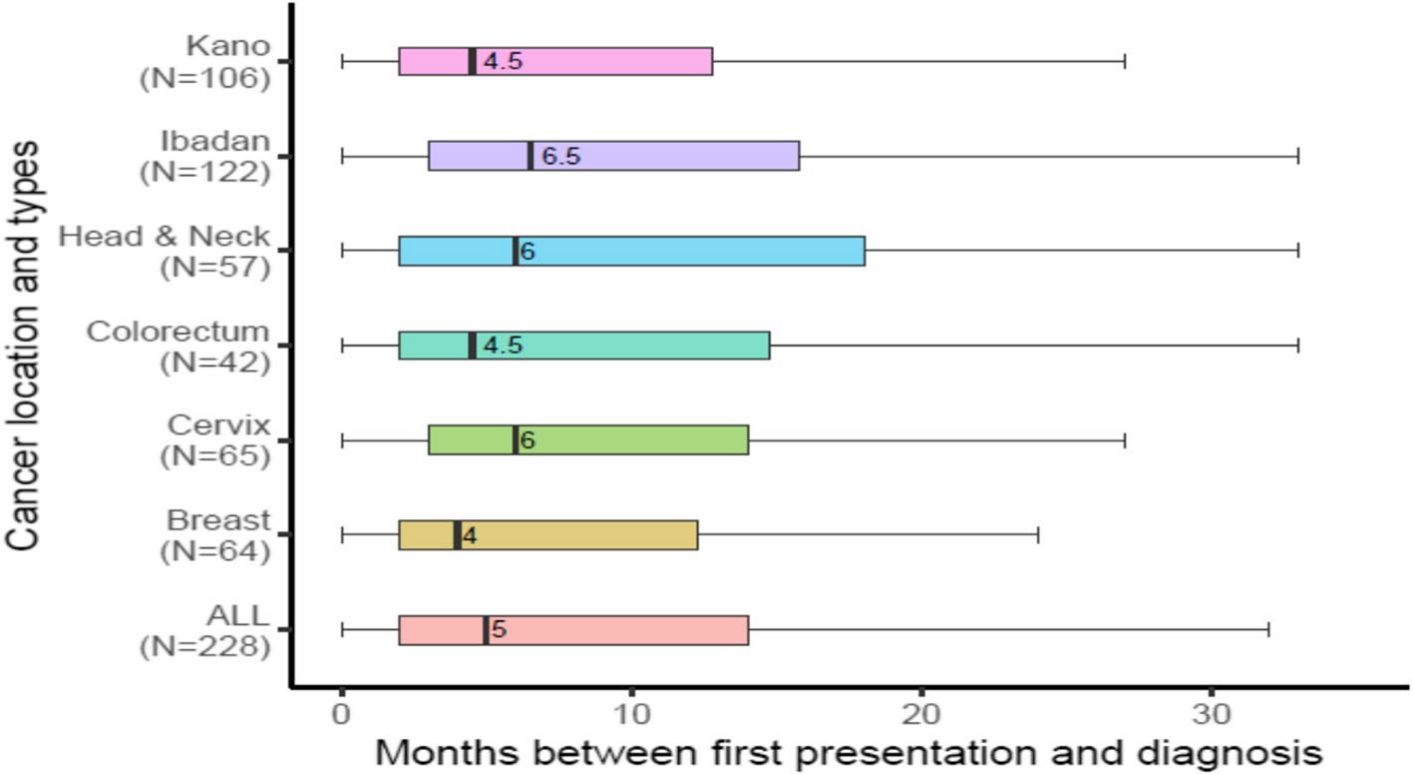
Duration of delay between first presentation to a formal healthcare professional and diagnosis

The median time for patients to receive a cancer diagnosis after the first presentation of symptoms to a formal healthcare professional was 5 months (interquartile range 12 months) (Fig. 3). Again, patients with cervical and head and neck cancer had a longer median time to diagnosis post-presentation than patients with breast and colorectal cancer.

**Fig. 4:**
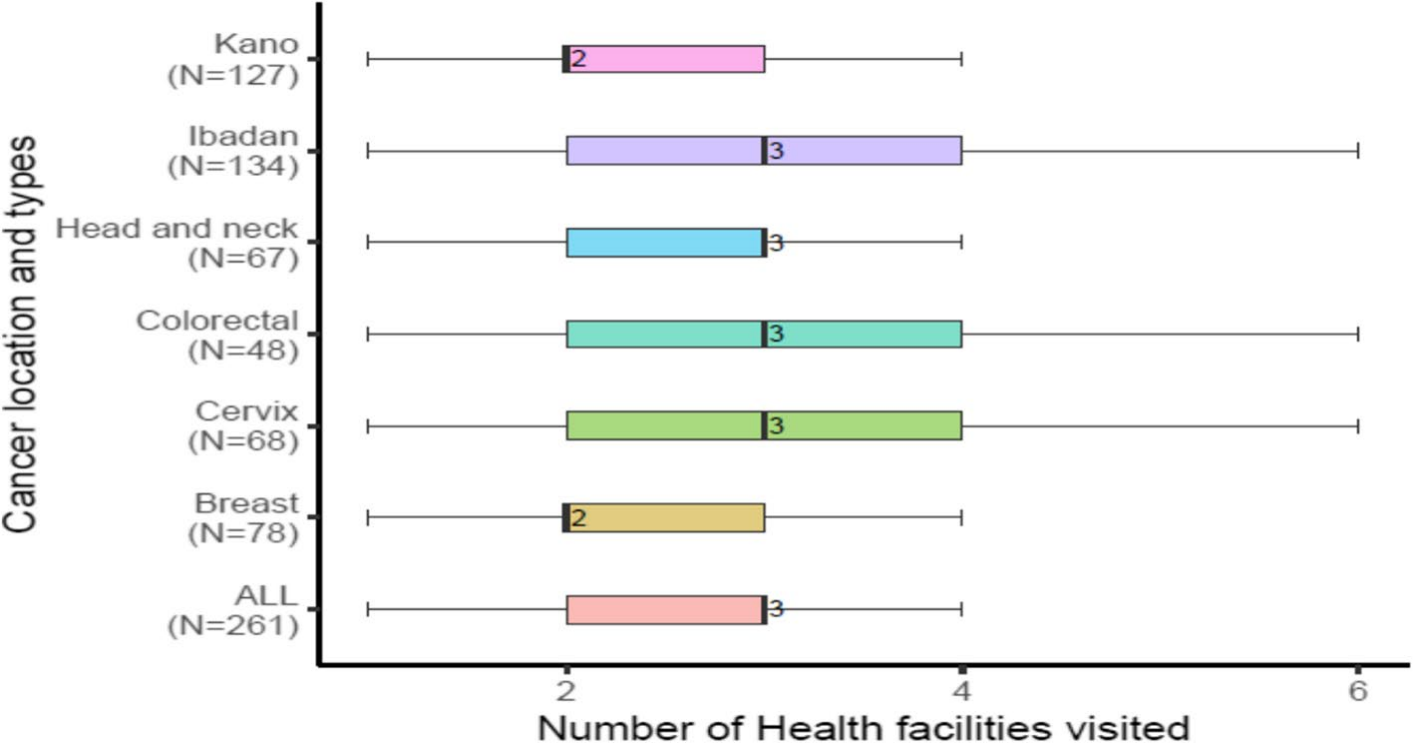
Number of health facilities visited between first symptom and diagnosis

Figure 4 provides information on the median and range number of healthcare facilities visited from the onset of symptoms to the eventual cancer diagnosis. Across all patients, the median number of health facilities visited before diagnosis in a formal hospital setting is three. Patients with breast cancer visited an average of two health facilities, whereas cervical, colorectal and head and neck cancer patients visited a median of 3 health facilities. However, the range was wide such that a quarter of patients with colorectal and cervical cancer visited four or more centres before receiving a diagnosis.

#### Reason for delays in cancer diagnosis

Reasons for delays in the cancer pathway to care consisted of patient, and health system and service provider factors.

### Patient factors

Patient factors contributing to delayed cancer diagnosis for most study participants are presented in Table 2. These factors include financial constraints, perceived non-seriousness of symptoms, lack of knowledge and misguided advice, awaiting the outcome of alternative therapy and commitments to family and spiritual beliefs. Overall, these patient-related factors highlight the complex interplay of psychological, social, and cultural factors that can influence the timing of cancer diagnosis.

**Table 2:** Key themes on patient factors relating to delay in cancer diagnosis

Financial constraint	*I didn’t have enough money, that was why I chose to go that hospital first. Charges there are cheaper than the Teaching Hospital. [In the chosen health facility] He [i.e. surgeon] gave me some test to do but I didn’t do them. I stopped coming to the hospital.…I had financial problem. I came back to the hospital after four years. (Female, aged 44 with cancer of the breast, Kano)*
Lack of knowledge	*If we had the knowledge about the disease*,* we probably would have come earlier. The government should sensitise the community about signs and symptoms of disease and encourage people to present early to the hospital. (Male aged 22 with cancer of the colorectum*,* Kano)*
Alternative therapy	*I went to tell a doctor and he said it means nothing*,* that I had done menstruation a long time ago*,* but the one I saw now*,* he said there’s no worry about it. So that was when we came home*,* we came to Ogbomosho; when we got to Ogbomosho*,* I heard these people advertising on the radio*,* these people that sells medication for Haemorrhoids on the radio*,* Marathon! I went to complain to them*,* and they gave me many medications*,* I was taking them*,* taking them*,* but I noticed I was still bleeding. So*,* I called them that I was bleeding*,* they said I should just continue with their medicines. (Female aged 78 with cancer of the cervix*,* Ibadan)*
Fear and Denial	*They didn’t do anything for me but asked me to go to Murtala Specialist Hospital. When I went there*,* they told me to come back the following week Friday. I didn’t go back because I was afraid they will remove the breast.…That was what people keep telling me. Even the health workers at the PHC told me that my breast will be removed. So I was scared and continue using the traditional medicine. (Female aged 18 with cancer of the breast*,* Kano)*
Social responsibility and commitments	*I think sometimes if you have other worries whatever they might be*,* you just tend to neglect……You tend to neglect your own health without actually meaning to because technically*,* while you are in that space*,* you are just focused on I need to sort this out*,* I need to do that and maybe even taking care of other people*,* you neglect yourself. (Female aged 42 with cancer of the breast*,* Ibadan)*
Role of Informal Caregiver	*…I have many children like you*,* your age.…They know*,* but they did not take me seriously. Then they brought me here finally. (Male aged 71 with cancer of the head and neck*,* Ibadan)*

#### i. Financial constraint

The most frequently cited factor was financial constraint. While financial barriers were acknowledged in the context of initial health-seeking behaviour, they became more pronounced following referral to the specialist health sector, where significantly higher out-of-pocket expenses, such as diagnostic tests, were incurred. The expense of hospital care, rather than initial consultations, posed the greatest barrier. Patients highlighted how the high costs of diagnostic tests and treatments discouraged them from seeking timely medical attention. Covering even basic hospital tests proved difficult for those struggling with daily survival needs, such as food and transportation.

Limited financial resources also delayed the collection of test results, with some patients spending spent significant time gathering funds from family and friends. Fluctuating costs relating to diagnostic procedures exacerbated the issue As a result, some sought care at less specialised facilities, delaying diagnosis. Only a few received external financial support for diagnostic procedures.

#### ii. Lack of knowledge and perceived non-seriousness

Many participants did not initially perceive their symptoms as serious, often viewing them as minor or common ailments that would resolve spontaneously. The absence of pain or significant discomfort further contributed to the perceived non-seriousness. For some, temporary relief from symptoms sometimes led them to postpone seeking medical help until symptoms resurfaced. Many participants sought advice from family and friends who reportedly gave false reassurance or advice to attend traditional or faith healers. Nihilism about treatment effectiveness also emerged as a reason to prevaricate.

#### iii. Alternative therapy

Some participants lost valuable time waiting for therapies such as over-the-counter medicines, traditional herbs and spiritual healing to take effect. Many participants frequently engaged in alternative treatments for conditions they believed to be infections or common illnesses, which contributed to delays in cancer diagnosis. They commonly used various symptom-relief methods, including pharmacy drugs, traditional herbs, and spiritual healing practices. Some individuals relied on home-administered injections, antibiotics, and creams, or prioritised traditional remedies over seeking formal medical care. Despite the lack of symptom improvement, several continued to use these alternative therapies, further postponing the identification of cancer symptoms. Faith healing was also sought, reflecting local beliefs in supernatural causes of illness, contributing to extended diagnosis delays.

#### iv. Fear and denial

While some participants did not recognise the importance of serious symptoms, others recognised them all too well, resulting in fear and denial. Fears were multifaceted, ranging from concerns about poor prognosis to anxiety about surgical procedures and adverse outcomes like childlessness.

Fear was a dominant theme in interviews with patients with breast cancer in particular, with most patients expressing fear of mastectomy. Also, patients reported fears related to health professionals and specific health facilities, as well as apprehension about sample collection for biopsy.

#### v. Social roles and family commitments

Participants’ delayed diagnoses were influenced by their social roles and commitment to family. There were instances of distractions within their social groups, such as illness and death in the family, causing them to overlook their health concerns and miss clinic appointments. Spousal influence, particularly for women who relied on their husbands’ support for medical decisions, further prolonged delays and hindered timely medical intervention. This lack of social support and alignment in the perception of the seriousness of symptoms between spouses contributed to the delayed diagnosis experienced by participants.

#### vi. Role of informal caregivers

Patients’ narratives highlighted informal caregivers’ significant role in facilitating patients’ diagnostic processes. Many participants reported delays in accessing medical care due to unavailability or passiveness of their caregivers, who were usually adult children of older participants. The proximity and availability of caregivers due to work commitments were also identified as contributory factors to delays in diagnosis and treatment. Despite patients being aware of their illness, diagnosis and treatment were often postponed due to caregivers not perceiving the situation with the same urgency. In some cases, caregivers delayed care by withholding consent for medical interventions.

### Health system and service provider factor

Some participants mentioned health system and service provider factor as reasons for delayed cancer diagnosis (Table 3). These factors include misdiagnosis and inconclusive results, delayed biopsy-histology results, health worker industrial strike action, attitudes of healthcare workers, administrative processing issues, infrastructural deficiencies, and distance to health facilities.

**Table 3:** Key themes on health system and service provider factors relating to delay in cancer diagnosis

Misdiagnosis and inconclusive results	*When I got to general hospital, I was told to go and do abdominal scan, and after I did the abdominal scan, they said they didn’t see anything. It was where I went to do the scan that someone asked me what my complaint to the doctor was before he said I should go for abdominal scan; I said I was bleeding, he said there was nothing, I then went back home. After that my blood was taken and they said I was having infection, that was what I was treating and it wasn’t going, so I stopped going to general and went to the private hospital we were using……Even after going, they still couldn’t get what was the issue with me…they were just telling me it was infection, but it wasn’t infection. (Female aged 50 with cancer of the cervix, Ibadan)*
Delayed biopsy-histology results	*I was examined by a Doctor. The doctor said they needed to do a test for me to know the type of sickness I have. After they did the test*,* I waited very long for the result and every time I called*,* they told me the result is not yet ready. So*,* I got tired and stopped asking them. (Female aged 48 with cancer of the cervix*,* Kano)*
Limited access to healthcare specialities and obstacles in the administrative process	*I’ve been referred since June and I’ve been coming…. and it has always been a kind of… this week we are having a meeting. Next week*,* there is a course we’re going for ehh there is an engagement. From that June there is… strike. So*,* at the end of the day*,* we had the surgery in September ending. Before we had the surgery*,* the thing has… metastasised. (Male aged 38 with cancer of the head and neck*,* Ibadan)*
Hospital equipment and infrastructure	*You know a lot of time*,* the engine in… may not be working. Sometimes we want to do a test and they tell us an engine is not working. There was a time we were asked to go and do it outside when the engine was not working. So*,* things like that can cause it…There was a month where for about…15 days*,* we were told there was no light in… (Female aged 45 with cancer of the head and neck*,* Ibadan)*
Distance to the health facility	*…They told me to come and collect it [i.e. the result] in fourteen days*,* I’m the one that [did not go back]… The place is far from my base*,* so since they discharged me in the hospital*,* I just took time before going and*,* before collecting it. (Female aged 41 with cancer of the cervix*,* Ibadan)*

#### i. Misdiagnosis and inconclusive results

Participants experienced delayed cancer diagnoses due to misdiagnosis, which was a prevalent health systems-related factor reported across all cancers and study sites. Symptoms were dismissed as “nothing” or misdiagnosed as non-cancer illnesses. Table S1 in the supplementary file lists the itemised mentions of situations where cancer symptoms were dismissed as “nothing” or misdiagnosed as non-cancer illnesses for the four cancer types. Participants in both sites reported that cancer of the cervix, breast, and head and neck cancers were often misdiagnosed as unspecified infections. Colorectal cancer was often misdiagnosed as haemorrhoids, ulcers, appendixes and typhoid. The dismissed symptoms and wrong diagnosis occurred in formal public and private health facilities. There are also a few instances of misdiagnosis from multiple health facilities undergoing various tests, each with varied and contrasting conclusions and, sometimes, receiving inconclusive results that discouraged further attendance at health facilities.

#### ii. Delayed biopsy-histology results

Participants, especially those from Southern Nigeria, frequently mentioned delayed biopsy/histology results as a major cause of delayed diagnosis. Internal human resource challenges, such as staff shortages or laboratory staff illness, further caused delays in processing biopsies and delivering results. They also mentioned that inefficient result transmission systems between health facilities contributed to prolonged waiting periods for test results. In extreme cases, delays were so severe that patients abandoned the diagnosis process altogether, leading to significant diagnostic delays.

#### iii. Limited access to healthcare specialties and obstacles in the administrative process

Participants identified several factors contributing to delays in the cancer care pathway, specifically relating to timely access and care from healthcare personnel in clinics. These factors included limited access to specialists, poor communication between patients and physicians, unprofessional attitudes of healthcare workers, and disruptions in clinic schedules. Some participants reported being unable to see their healthcare specialist and were rescheduled for later dates due to the absence of a replacement doctor. Inadequate patient-physician communication further hindered care, with participants expressing frustration over insufficient information despite their inquiries. Additionally, in both study sites, participants highlighted administrative barriers, such as difficulties in securing timely appointments, mishandling of medical files, delays in insurance processing, and inefficiencies in payment systems.

#### iv. Hospital equipment and infrastructure

As a health systems factor, delayed test results were driven by inadequate infrastructure, leading to ineffective service delivery. Participants gave examples relating to interruptions to electricity supply, faulty machines and inefficiencies in the result transmission system between health facilities. Equipment breakdown at hospitals, particularly in Ibadan, caused delays in diagnosis as patients had to seek private services due to the unavailability of diagnostic equipment, which could be costly. Issues such as poor electricity supply contributed to equipment inoperability, further prolonging the diagnostic process and impacting patients’ access to timely care.

#### v. Distance to health facilities

Distance to the health facility emerged as a factor contributing to delayed diagnosis, particularly noted by participants in Ibadan. The distance between participants’ residences and the health facility hindered their ability to promptly return for test results or seek diagnosis, as travelling was perceived as burdensome. Lack of familiarity with the area compounded the issue for participants who were not from the vicinity of the health facility, making it challenging to navigate and seek assistance locally.

## Discussion

### Delay in seeking care

Our study confirms long delays in accessing cancer care across four common curable cancers in the second and third largest cities in Africa’s most populous country. The median time from first noticing a symptom to diagnosis was 12 months (365 days) across the four cancers. This is similar to the interval observed in Tanzania, where the median delay was 358 days [23]. The 12-month delay in our study was all the more concerning because it does not include delay from diagnosis to treatment. There was a notable difference in overall delay between different cancers, with head and neck having the largest overall delay (15.5 months) across the four cancers, followed by cervical cancer (9.5 months). Our findings are in accord with systematic and meta-analytical reviews showing the interval from first symptom to diagnosis is 1.5 to 4 times longer in low-income countries than in high-income settings [24, 25].

The problem with the late presentation is typically ascribed to a delay in health-seeking. Remarkably, we find that delay within the formal health sector is considerably longer than delay to presentation across all four cancers. This corroborates previous findings from East Africa [23, 26]. Whereas the median interval of three months was reported between the first notice of symptoms and presentation to a healthcare professional, the median between presentation and diagnosis was five months.

Our study also showed evidence of “churning,” a situation where cancer patients move around multiple health facilities in search of solutions [23]. From symptoms to diagnosis, they visited a median of three and up to six health facilities. The reality of churning in cancer care pathways is a key driver of the out-of-pocket expenditure burden experienced by mostly poor cancer patients in poor countries.

### Causes of delay

The study findings align with two of the Aarhus framework’s contributing factors to delay diagnostic and treatment, patient and health systems factors. Out of pocket expenditure, the role of informal caregivers, misdiagnosis and problems with histology emerged as prominent themes. Our data show that less than 5% of participants had health insurance. Therefore, it is not surprising that finance was frequently mentioned, especially after referral to the specialist system. However, finance was by no means the only barrier. We encountered many patient and service barriers reported in previous studies in high- and low-income countries. Most participants in the study had a formal education but appeared to have low health literacy concerning cancer symptoms. Such people rely on family, social and religious networks for information and support. Our data shows that such networks were often a barrier to care; the importance of symptoms was downplayed, patients were misdirected to a traditional healer or health seeking was mis-represented as a sign of insufficient religious faith. The role of traditional healers is controversial; some studies suggest they are consulted only rarely for health matters [27], while others suggest that they are consulted frequently in the context of cancer symptoms, and that such consultation is associated with worse prognosis [24]. Yet, others find that traditional healers are consulted when patients encounter delays *after* referral to health services [23]. There were also health system factors, which ranged from diagnostic problems to lab testing and result collection delays, attitudes of health workers, bureaucracy, and distance to health facilities.

### Implications of our findings

Delayed cancer diagnosis results in poor outcomes [5, 6, 17], especially in sub-Saharan Africa, where therapies to treat advanced cancer are seldom affordable [28]. The overarching implication of our findings is that this problem cannot be ignored. Governments and donors around Africa are investing money into the development of advanced facilities, including linear accelerators [14, 15]. In Nigeria, the federal government recently committed to such investments through the oncology initiative which seeks to enhance oncology care through strategic medical investments [8]. Yet most people cannot access these facilities because they cannot afford them. Indeed, a recent consensus statement published in *Nature Medicine* identified expedited presentation as the top priority for cancer care research in LMIC [29].

Our findings can help in identifying priorities. First, our finding is that most delays occur within the health service, so attention should be focused on improving service pathways. A particular barrier arises at the point of histology. We advocate first education of primary care clinicians as to where specialist services, including access to inexpensive histology, are available to avoid churn and the out-of-pocket expenses it entails. Second, histology services should be audited, and the audit results be made publicly available. Third, states in Nigeria should consider establishing centralised low-cost histology services as in parts of Kenya. Fourth, studies on methods to reduce the cost of histology should be undertaken, including using Artificial intelligence (AI) for analysis of samples.

We note that failure to refer was an important source of delay in our data. This finding is consistent with the extensive literature on the quality of primary care consultations in LMIC [30]. The obvious remedy here is education, which has improved cancer referral practice in UK [5]. Education modules should be tailored to each country’s needs and follow evidence-based principles of continuing professional development, for example, using inter-active learning, enhancing motivation and re-enforcing knowledge. The modules should be evaluated and approved for continuing professional development to become scalable and sustainable. Since many people have low cancer literacy and/or considerable trepidation regarding health services, we suggest implementing navigation assistance, which has proven successful in other (non-cancer) contexts [31, 32], to provide emotional and logistic support to those who need it.

Regarding the issue of healthcare seeking, we note the importance of community networks in our data and the broader literature [33, 34]. This suggests that public education should include a community focus to tackle some of the barriers we have documented. First, we think that education regarding cancer should be included in continuing education for Community Health Workers – a policy already enacted in Kenya. Second, many studies have shown that it is possible to engage respectfully with traditional African healers to encourage referrals for people who need allopathic care [35, 36]. Such engagement should include awareness of serious symptoms of diseases like cancer and tuberculosis. Third, we note that faith healing was pursued, reflecting local religious beliefs in supernatural causes of illness, which can delay care, and, again, there should be respectful engagement with religious leaders to alter practice, as seen in the Ebola epidemic in West Africa [37].

### Strengths and weaknesses

Our study has the strength of covering four cancers, whereas previous studies have tackled only one or two [38–42]. Our study has the further strength that it combines measurement of delay with qualitative data to shed light on the reasons for those delays across two culturally very different areas in Nigeria. We found similar patterns across these cities, suggesting that our findings are broadly applicable. We designed our study to maximise recruitment of participants for whom interventions with curative intent are unrealistic while complying with strictures on home visiting. However, this strength came at the cost of a limitation because we do not have data on the diagnosis to treatment interval. Another limitation arises from the strike action of the National Association of Resident Doctors during the study. Fortunately, the strike was relatively short, lasting 17 days, from July 26 to August 12, 2023. Our data collection ran for 26 weeks, as mentioned above. Thus, the strike could explain little of the 15.5-month delay observed, for example, with respect to head and neck cancer. We followed the Aarhus declaration to ensure compliance with current best practices. However, the method has limitations since it is based on recall [43]. We tried to minimise recall bias by using calendar methodology and asking participants about other life events to assist recall. We may have missed some cases, such as those who died without presenting or presented as an emergency, but again, this bias would lead to an underestimation of delay. In addition, it is possible that patients experiencing financial hardship were not captured in our study due to a lack the financial means to seek care in secondary or tertiary health facilities.

## Conclusion

Addressing the challenge of delayed diagnosis is important for successful response to cancer treatment and survivorship in LMICs. We examined the duration and reasons for the delayed presentation and diagnosis of four common and treatable cancers in two regions of Nigeria.

## Supplementary Information

Supplementary Material 1.

## Data Availability

This study data cannot be shared publicly because of ethical requirements to protect the confidentiality of our participants. To request access to anonymised data, please contact the corresponding author at the University of Birmingham (Dr Patricia Apenteng: p.n.k.apenteng@bham.ac.uk).
