# Supplementary material for: Diagnosis of cancer in the South and North of Nigeria: duration and causes of delay
Source: BMC Health Serv Res. 2025 May 21;25:738. doi: 10.1186/s12913-025-12707-8 (PMC12093698; doi:10.1186/s12913-025-12707-8)
Supplement: Supplementary file 1 — Supplementary Material 1. [file 12913_2025_12707_MOESM1_ESM.docx]

Supplementary file

1. Supplementary tables

Table S1: Instances of misdiagnosis by cancer type, according to study site

| **Cancer type** | **(Mis)diagnosis (Ibadan)** | **(Mis)diagnosis (Kano)** |
| --- | --- | --- |
| Breast (11) | “Nothing” (6); “Infection” (2) | “Nothing” (3) |
| Cervix (18) | “Infection” (9); “Nothing” (1); “Fibroid” (2) | “Nothing” (1); “Infection” (1); “Ulcer” (1); “Vaginal infection” (1); “Miscarriage” (1); “Fibroids (1) |
| Head and Neck (3) | “Nothing” (1); “Infection” (1) | Nothing (1) |
| Colorectal (22) | “Haemorrhoid” (3); “Ulcer” (2); “Nothing” (1); “Appendix” (1); “Irritable Bowel Syndrome (IBS)” (1); “Typhoid fever” (1) | “Ulcer” (4); “Haemorrhoid” (2); “Nothing” (2); “Malaria” (1); “Dysentery” (1); “Appendix (1); “Typhoid fever” (1); “Heart problem” (1) |

**B. Additional quotations supporting themes**

Box S1. Additional quotations for themes on patient factors relating to delay in cancer diagnosis

| Financial constraint | *The first thing is money, because when I went to the lab, they said the first test was around ninety something thousand, so, I was like, “what! it’s as much as that…?” So, …I now left the hospital… That was when I went back to West Wind [another hospital] and they told me to go for some tests, so all those tests… when I went for it, they said it’s huge amount of money; so, the money was much for me… My savings is not even up to that… (Female, aged 24 with cancer of the colorectum, Ibadan)*  *We barely have enough to eat now. …We have serious financial problem…. Sometimes we come to the hospital without breakfast because we don’t have the food to eat. Even transport money we have to look for assistance. (Female, aged 67 with head and neck cancer, Kano)*  *…When I first came and they said I should look for money, there was no money, you understand? So, there was no money I would bring, and all the children are not buoyant. (Female, aged 63 with cancer of the cervix)* |
| --- | --- |
| Lack of knowledge | *If I had known, from the time I started stooling, and there was blood in it, and I noticed it, I would have known. I thought it was haemorrhoid. (Male, aged 30 with cancer of the colorectum, Ibadan)*  *If I had known that the traditional medication doesn’t work I will have come earlier. (Female, unknown aged cancer of the breast, Kano)*  *I noticed that there was a swelling in my breast; I was touching it thinking it was a boil coming up. I decided to be applying Shea-butter to it; normally you apply it on a boil so that it can bring out an opening so that it can burst. So I discovered that there was no opening and it pained me a little for a while, then it will stop. So I tried to keep it to myself then, but I later told one of my neighbours, she gave me Shea butter and Ori-atu I used to apply them but it didn’t stop. So I went to my area mother because she is a little learned, so she said there was something called lumps, I’ve not heard about it before, so I was like lumps…she said I should go to the nearest hospital which is a private hospital, that they will explain it to me better. (Female, aged 33 with cancer of the breast, Ibadan)* |
| Alternative therapy | *I was just there. I was taking antibiotics. Then I was also applying the cream they brought for me to rub, let me see if it will work. So, when I felt that none of these things were working, I now went to the hospital. (Female, aged 47 with head and neck cancer, Ibadan)*  *As I told you earlier we had the intention to come to the hospital (My husband and I) but our elders in the house insisted we try traditional medicine first. … I have started using traditional medicine and thinking it was effective. (Female, aged 42 with cancer of the breast, Kano)* |
| Fear and Denial | *He [doctor] told me that he will give me some injection and take some tissues from the breast for test. People had been telling me not to take any injection. …He therefore asked me to come back next week with any of my relative. But I didn’t come back for the biopsy till after about five months. (Female, aged 70 with cancer of the breast, Kano)*  *When we got to Oluyoro and gave them the letter, they said they do not have the doctor and the machine and maybe I should go to UCH. I said, I’m always scared of UCH and its trouble of going up and down. So, we went home and since then, I have been using the drugs I said was prescribed and they worked. But… (Female, aged 53 with cancer of the colorectum, Ibadan)*  *…When someone says, you are sick, you tell yourself that you are not, even the bible says that. You tell yourself you are strong. (Female, aged 30 with cancer of the breast, Ibadan)* |
| Social responsibility and commitments | *My husband immediate younger sibling was ill, and we were running to and from; later when the sibling died, we had to go to our hometown, because the children were young, we went home. It was when we came back that we gave more attention to this… (Female, aged 54 with cancer of the cervix, Ibadan)*  *We did not go back after they told us to do the tests because my child got sick (Female, aged 58 with cancer of the colorectum, Kano)* |
| Role of Informal Caregiver | *We then noticed a swelling close to my neck. They said they will remove it but my mother refused to consent. She said I may die and I am her first daughter. So, I continue with my herbal medication. But it keeps growing. (Female, aged 76 with cancer of the breast, Kano)*  *We were supposed to go back the following week, the way my family is, I’m really the only available person because I’m an entrepreneur, I’m not in full time practice like so, I’m the only one that has time, like so the week he was supposed to be in surgery, I was on field for a particular job, so I couldn’t come. (Informal caregiver of male, aged 20 with cancer of the head and neck, Ibadan)* |

Box S2. Additional quotations for themes on Health system factors relating to delay in cancer diagnosis

| Misdiagnosis and inconclusive results | *I think in 2020, I went to the hospital. I went to one centre, so the doctor checked it, but she told me there was nothing. So, after then I went to the hospital again, but the doctor was not there, you know general hospital doctors don’t come on time, so the nurse checked it. She told me there was nothing there, but I told her I was feeling something. She told me there was nothing, so I went back home. (Female, aged 43 with cancer of the breast, Ibadan)*  *Participant: I never imagined it is going to be serious. I couldn’t eat much during the festival. I went to Murtala Muhammad Specialist Hospital MMSH on 27th April 2023 I had a blood test and the result shows Malaria. I was given some malaria drugs though I told the doctor that I don’t feel feverish I only had stomach pain he still insisted it was malaria. I came back home and was using the drugs he gave me. The following week we travel to Kaduna for an occasion and I came back to Kano with fever. I went to a chemist because it was late in the night and I was told to have malaria. He gave me some injections and antibiotics (Amoxil). After 3 days, the stomach pain persist. I returned to that chemist and he said it was dysentery.*  *Interviewer: He told you it was dysentery right?*  *Participant: Yes that the malaria caused it. I used to go to toilet 10 times and it was mostly mucoid stool. He gave me some intravenous infusion saying that it will cure the dysentery. There was no improvement so I went to another hospital. … All these happened within 8 to 9 days. I would have gone back to MMSH but lack of time made me went to Primary Health Care. The doctor said I should go and do a scanning and the result shows appendix and my intestine has a hole. He was shocked seeing that result and he referred me to MMSH because it is closer to me. The next day I went to MMSH straight to Emergency section. I was told to come back the following day to see a senior doctor. The doctor saw the result and interpreted differently from what the other doctor said. He ordered me to do another scanning inside the hospital and before I could come back with the result he has closed for the day. I showed it to the next doctor on duty. He said it is a small problem in my abdomen and prescribed some drugs to me. The pain still persists. Then, my sister’s husband called his in-law, a medical doctor to come and see my condition. He came to our house and wrote another test to be done. He did another blood test and said it is typhoid and malaria plus infection. He treated me with intravenous medication and left. (Male, aged 30 with cancer of the colorectum, Kano)* |
| --- | --- |
| Delayed biopsy-histology results | *…They said I would collect the result after two weeks, I got there in two weeks and they asked me to come back in another two weeks, after another two weeks, after another two weeks, they said I should come back in another two weeks. When I went there in another two weeks, I started shouting on them that do they want to kill me? … I went to report them to the Matrons… they later discovered that they are yet to take the sample that was removed from my body out of the theatre. (Female, aged 40 with cancer of the cervix, Ibadan)*  *We waited for almost 3 weeks and the result was not out. So, I went to the hospital and told them, they are asking us for the result of biopsy at AKTH. Then, they said the person at the Lab was sick. So, they took another biopsy which take 10 days for the result to be out. (Male, aged 18 with cancer of the head and neck, Kano)* |
| Limited access to healthcare specialities and obstacles in the administrative process | *Today will be the 3rd time. On the 20th of July, I learnt that a strike commenced, the resident doctors so that’s the first one. I was given an appointment date for…June, July. I came in June but there was an ongoing strike. Then I came in July, July 20th exactly and they said another strike commenced. (Female, aged 59 with cancer of the cervix, Ibadan)*  *It was at the scanning that they say that there is a problem. I took the result to the doctor but he tore the result after reading it. He now send me to Buhari Specialist Hospital to do another test which I could not do because they are doing renovation. (Female, aged 53 with cancer of the breast, Kano)*  *Where I was asked to go for the test, we were unable to generate a [health insurance] code at the place I was asked to go for the test and I was told this should be done a day before I will do the test. We were unable to generate a code in NHIS till the day ended and I couldn’t do the test without it. …I had to come back and told them I was unable to do the test so they fixed another date. Maybe first of December because there were already a lot of people waiting. (Female, aged 58 with cancer of the breast, Ibadan)* |
| Hospital equipment and infrastructure | *…Assuming the first time she went to FMC hospital, they had solved it and it did not have to stay long like this. …They didn’t have the machine. (Female, aged 49 with cancer of the breast, Ibadan)*  *I went to Murtala Specialist Hospital. They said I need blood transfusion but no bed for admission. So we went to another General hospital but also no bed space for admission. I went to another private hospital but they told us to pay N50,000 before they will admit us, which we don’t have. We went back home. (Female, age unknown with cancer of the breast, Kano)* |
| Distance to the health facility | *The reason why I didn’t come at that I’m a bit far. …I don’t stay permanently in Ibadan. …I’m in Abuja. (Male, aged 53 with cancer of the head and neck, Ibadan)*  *We are not from here and we don’t know anybody here, you if we know someone here…when we later asked, we were then directed to someone who might help us, because we are not from here, we don’t have a house here… (Female, aged 64 with cancer of the cervix, Ibadan)* |

C. Data collection instrument.

**DELAY PRESENTATION OF CANCERS IN NIGERIA**


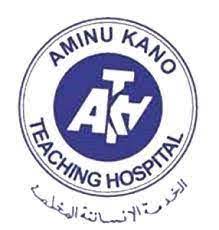
**



**


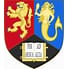


**University of Birmingham**

**PATIENT PARTICIPANTS PROFORMA**

**SOCIO-DEMOGRAPHIC CHARACTERISTICS**

ID NUMBER:

Age: Gender:

Occupation:

Type of Cancer:

Are you married: YES NO

Tribe Religion

Highest education: No Formal Primary Secondary Tertiary

Currently working (If unsure, worked in the last week)? YES NO

How do you plan to pay for your healthcare?

Self Family and Friends Health Insurance All of the above

Not sure

***PATIENT INTERVIEW GUIDE***

**Patients’ accounts of their journey from first noticing symptoms to initiating treatment**

**Stage 1: Awareness and accessing care**

1. Looking back what was the very first thing you noticed?

Probes:

- Blood in stool, diarrhoea and other changes in bowel habits (Colorectal)
- Breast lump, discharge and/or tenderness (Breast)
- Persistent sore throat, lump/ ulcer in mouth, hoarse voice, voice change, tooth ache, nasal blockage, bleeding from the nose (Head and Neck)
- Abnormal vaginal bleeding (Post coital bleeding, intermenstrual bleeding, post-menopausal bleeding), vaginal discharge (Cervical)

1. When was this? (use ‘calendar’ and best estimate a plausible range)

Probes

- Was it last year?
- Do you remember anything else that was happening around that time?

1. When you first noticed these things did you think it might be serious? *If YES please go to Question 5*
2. When did you first think it might be serious? *(How many weeks/months after you first noticed a bodily change?)*
3. Can you tell me why you thought it was serious?

*Probes - Explain*

1. Before coming to the formal health centre:

6a. who have you discussed with?

6b. Did you discuss it with any of following or their intermediary?

Probes -

- 1. Did you speak to a Chemist/PMV? If yes, why? In what way did you think they could have helped?
  2. Did you speak to a pharmacist? If yes, why? In what way did you think they could have helped?
  3. Any other community health worker? If yes, why? In what way did you think they could have helped?
  4. Did you consider a traditional healer or attend a traditional healing centre? If yes, why? In what way did you think they could have helped?
  5. How about a religious leader or faith healer? If yes, why? In what way did you think they could have helped?
  6. Did you discuss it on social media or consult internet? If yes, why? In what way did you think it could have helped?

If possible, probe all of the above and encourage patient to speak freely.

If they mention several of the above ask – Which was most important. Try to find out when in the patient’s journey they connected with this person.

1. When you first saw a health professional, was it any of the following type
   1. Primary Healthcare Centre, General Hospital or Specialist Centre
   2. Private or Public Health Facility
   3. Doctor, Nurse or other healthcare worker
2. Why did you choose the ‘health facility’ you chose?

Probe- please probe convenience, cost, proximity, confidence, religious obligations, family considerations, quality, social media, other options.

1. When did you first see this doctor or nurse following the bodily change? (use ‘calendar’ and best estimate a plausible range)
2. What, if anything, hindered you from seeing the health care provider on time?

Here you should explore the causes of any delay –

Probes

- *Was it cost? Influence of people - who? Lack of knowledge? Self-denial?*

1. What may have helped you avoiding delay in seeing the health care provider?

**Stage 2: Clinical evaluation, diagnosis and staging**

1. Can you tell me what happened at the first clinic or hospital where you presented your symptoms?

Probes (***if not diagnosed at the first hospital of call***):

- Did the person examine you?
- After examination, what did the person say?
- Were you satisfied with the services at your first visit?
- Did the person refer you? – If so where to?

13. Where did you go next?

- Time between each clinic visited
- (Quantify in weeks/months/years, using previous reference points and anything else that was happening in the patient’s life at the time)
  - - Was it 2 months later?
    - That would be about (name the month) then?
    - Was there anything happening in your life around that time?
- Explore reasons for delay at each referral stage (e.g., lack of funds; belief system – cultural & religious; fear of the unknown; inaccessible hospital; transport etc.)

Repeat question (13) above for each referral or healthcare provider the patient visited until they received a diagnosis.

14. After coming in contact with a formal health facility, did you seek healing elsewhere?

Probe: Traditional healer, faith healer, faith leader, revival center or healing home

15. Can you tell me about the biopsy or tissue sample? This was when a tissue sample was taken from you.

- - Where was this done? How many biopsies did you do?
  - How did you feel about it?
  - How long did it take to get the results from the biopsy or tissue sample?
  - How long did it take to get the results of the biopsy/ tissue sample to the doctor?
  - Was there a wait time from the time you were told that you needed biopsy or tissue sample taken? (Quantify in days/weeks/months using earlier probes)
  - Was cost/money an issue?

16. When was the information about the histological diagnosis given to you by your doctor? (use ‘calendar’ and best estimate a plausible range)

Probes:

- Perceived reasons of delay in diagnosis (e.g., personal delays in diagnostic test [funds], clinical delay [bad laboratory], booking/logistics delay, attitude of involving health workers etc.)

**Stage 3: Access to treatment**

17. Were you informed that you would receive treatment for your condition?

18. When were you informed that you would receive treatment for your condition?

18b. What was the first treatment you received and when did you receive this?

- Probe Period of delay between the diagnosis and initiation of treatment

Reasons for delay of initiation of treatment (e.g., lack of funds; belief system – cultural & religious; fear of the unknown; inaccessible hospital; transport etc.)

19. Any other experience on the diagnosis and treatment of this condition that you want to share?

Thank you.
